# Global and quantitative proteomic analysis of dogs infected by avian-like H3N2 canine influenza virus

**DOI:** 10.3389/fmicb.2015.00228

**Published:** 2015-04-02

**Authors:** Shuo Su, Jin Tian, Malin Hong, Pei Zhou, Gang Lu, Huachen Zhu, Guihong Zhang, Alexander Lai, Shoujun Li

**Affiliations:** ^1^College of Veterinary Medicine, South China Agricultural UniversityGuangzhou, China; ^2^Key Laboratory of Comprehensive Prevention and Control for Severe Clinical Animal Diseases of Guangdong ProvinceGuangzhou, China; ^3^Harbin Veterinary Research Institute, Chinese Academy of Agricultural SciencesHarbin, China; ^4^State Key Laboratory for Emerging Infectious Diseases and Center for Influenza Research, School of Public Health, The University of Hong KongHong Kong, China; ^5^College of Arts and Sciences, Kentucky State UniversityFrankfort, KY, USA

**Keywords:** canine influenza virus, H3N2, global proteomic analysis, tandem mass spectrometry

## Abstract

Canine influenza virus A (H3N2) is a newly emerged etiological agent for respiratory infections in dogs. The mechanism of interspecies transmission from avian to canine species and the development of diseases in this new host remain to be explored. To investigate this, we conducted a differential proteomics study in 2-month-old beagles inoculated intranasally with 10^6^ TCID_50_ of A/canine/Guangdong/01/2006 (H3N2) virus. Lung sections excised at 12 h post-inoculation (hpi), 4 days, and 7 days post-inoculation (dpi) were processed for global and quantitative analysis of differentially expressed proteins. A total of 17,796 proteins were identified at different time points. About 1.6% was differentially expressed between normal and infected samples. Of these, 23, 27, and 136 polypeptides were up-regulated, and 14, 18, and 123 polypeptides were down-regulated, at 12 hpi, 4 dpi, and 7 dpi, respectively. Vann diagram analysis indicated that 17 proteins were up-regulated and one was down-regulated at all three time points. Selected proteins were validated by real-time PCR and by Western blot. Our results show that apoptosis and cytoskeleton-associated proteins expression was suppressed, whereas interferon-induced proteins plus other innate immunity proteins were induced after the infection. Understanding of the interactions between virus and the host will provide insights into the basis of interspecies transmission, adaptation, and virus pathogenicity.

## Introduction

Waterfowl are natural reservoir hosts for influenza A virus, which maintain a vast viral gene pool and pose a continued threat to humans and other mammalian species (Webster et al., 1992). The unique agriculture practice and ecological systems in China and other eastern Asian countries have provided ample opportunities for avian influenza virus (AIV) to cross species and cause sporadic infections in these mammalian hosts (Su et al., 2014a,b,c). Due to the close contact with humans and frequent interactions with wandering animals, poultry and sometimes wildlife, companion pets (cats and dogs) have been considered as potential “intermediate hosts” for the interspecies transmission of zoonotic pathogens to humans.

Dogs are susceptible to multiple subtypes of influenza A viruses. Interspecies transmission from human to dog or from avian to dog has been repeatedly observed. Historically, dogs have been reported to be infected with human seasonal H3N2, pandemic 2009 H1N1 viruses, equine H3N8, highly pathogenic avian influenza virus (HPAI) H5N1, lowly pathogenic avian influenza virus (LPAI) H9N2, and more recently, AIV H10N8 (Crawford et al., 2005; Songserm et al., 2006; Sun et al., 2013, 2014; Su et al., 2014b,d; Yin et al., 2014). Most of these belong to sporadic infections and the viruses could not become efficiently transmitted among dogs. One of the exceptions was the epizootic caused by an avian-origin H3N2 canine influenza virus (CIV). This was first reported in 2008 among dogs being treated at veterinary clinics in South Korea (Song et al., 2008). The H3N2 CIV infection could be further traced back to 2006 when a highly related virus was isolated in a sick pet dog in China (Li et al., 2010). This virus has spread to other canine populations in eastern Asia and even further to domestic cats (Lin et al., 2012; Su et al., 2013; Sun et al., 2013; Song et al., 2011). Serological findings of retrospective surveys suggested that the H3N2 virus might have been circulating in canine populations in China for some time prior to overt or reported epizootics (Zhao et al., 2011; Zhang et al., 2013). Thus, available information has revealed that this H3N2 virus has become established in canine population in these countries.

Introduction and establishment of an avian H3N2 influenza virus in dogs have caused continuous prevalence in this host species in Asia, which also raised concerns whether it could become a sustained risk to the public and the veterinary health. However, how did this avian-origin virus successfully establish infection in the new canine host was unclear.

Many studies on the molecular basis for AIV to infect and to adapt in mammalian species focused on viral genetic changes, i.e., various non-synonymous substitutions occurred in the viral genome such as Q226L and G228S in the hemagglutinin (HA) (Connor et al., 1994), and E627K, M147L (Wang et al., 2012), K339T (Liu et al., 2013), I588T (Foeglein et al., 2011), and K562R (Song et al., 2014) in the PB2 protein. Studies focused on the host response and the differential gene expression kinetics upon infection majorly used mouse or *in vitro* cell culture as the models (Korth et al., 2013), and in recent years, ferrets were also used (Bruder et al., 2010; Camp et al., 2012; Leon et al., 2013). As the host of the newly established avian-origin H3N2 CIV, dogs served as a unique model for the study of interspecies transmission and adaptation of AIVs in mammals.

In this study, we conducted a global and quantitative proteomics to identify host proteins that were differentially expressed upon inoculation of the H3N2 CIV in dogs. We have identified 278 host proteins that were differentially expressed at different time points after inoculation. Selected up-regulated and down-regulated proteins were validated by quantitative realtime PCR (qPCR) and Western blot. Up-regulation of many interferon (IFN)-induced proteins and other innate immune proteins was identified. Down-regulation of proteins involved in apoptosis and cytoskeleton proteins was also identified. The role of these positively and negatively induced proteins in relation to pathogenicity and viral adaptation is discussed.

## Materials and methods

### Virus and viral inoculation

CIV, A/canine/Guangdong/01/2006 (H3N2), was isolated from a dog with respiratory symptoms at an animal clinic in Guangdong, 2006 (Li et al., 2010). Virus stock was propagated in 10-day-old embryonated chicken eggs and titrated using 1% horse erythrocytes. For *in vivo* virus inoculation, 2-month-old beagles (*n* = 9) were used for inoculated and control groups, respectively. These dogs were shown sero-negative by hemagglutination-inhibition (HI) assay for seasonal influenza viruses (H1N1, H3N2, and influenza B viruses) and for avian-origin CIV (H3N2). Inoculation was conducted intranasally with 10^6^ EID_50_ of the virus in 1.0 ml phosphate buffered saline (PBS), under light anesthesia by tiletamine–zolazepam (Virbac, 10–15 mg/kg). For the control group (*n* = 9), animals were mock inoculated with 1.0 ml of PBS. All animals, except for those euthanized for lung tissue collection, were monitored daily for body temperature, virus shedding and clinical signs for 14 days, and serum samples were collected at 14 dpi.

At 12 hpi, 96 hpi (4 dpi), and 168 hpi (7 dpi), two animals from each group were euthanized by pentobarbital overdose. Lung tissues were surgically removed and processed for detection of the viral replication, hematoxylin-eosin (H&E) staining, immunohistochemistry examination and iTRAQ analysis. The lung tissue samples from two dogs were analyzed individually. At 12 hpi, the lobus cardiacus of right lungs from a dog was homogenated in liquid nitrogen. At 4 and 7 dpi, lung tissues with lesions were sampled in the lobus cardiacus of right lung in inoculated group and the corresponding sites in the control group were chosen for sampling and processing. Other parts of excised lung tissues were collected for detection of the viral replication, hematoxylin-eosin staining and immunohistochemistry.

### Ethics statement

All procedures in the animal experiments were approved by the South China Agricultural University Experimental Animal Welfare Ethics Committee with a reference number of 2014–05.

### Histopathology and immunohistochemistry of the lungs of infected dogs

Lung tissues were fixed first in 10% formalin, followed by embedding in paraffin. Five-micrometer-thick (5 μm) slices were stained with hematoxylin-eosin and examined under an Olympus DP70 microscope (Olympus Corporation). A second set were used for immunohistology, using rabbit anti-influenza A virus nucleoprotein (NP) antibody (Serotech). Briefly, tissue sections were de-paraffinized and then fixed with 100% chilled-acetone for 2 h. The sections were incubated with 3% H_2_O_2_ for 15 min at 37°C before blocking with 5% bovine serum albumin (dilution in PBS) for 1 h. The blocked sections were then incubated with rabbit anti-influenza A virus NP antibody (1:1000 dilution) at room temperature for 1 h. The labeled tissue sections were stained with horseradish peroxidase (HRP)-labeled goat anti-rabbit antibody (Sigma-Aldrich) and alkaline phosphatase substrate, and then counterstained with hematoxylin QS (Vector Laboratories).

### Sample preparation, iTRAQ labeling, and peptides fractionation

Total protein samples were reduced and cysteine-blocked according to the protocol provided by the manufacturer (iTRAQ kit, Applied BioSystems). Samples were then 1:10 diluted in 0.5 M triethylammonium bicarbonate (pH 8.5). Each sample was treated with 1 μg trypsin (25 μl) and kept at 37°C overnight. After the samples were vacuum-dried, they were resuspended in 35 μl of 0.5 M triethylammonium bicarbonate (pH 8.5). The non-infected and infected samples were labeled with iTRAQ reagents 114 and 115, respectively. These labeled protein samples were pooled and then purified using a strong cation exchange column. The bound peptides were eluted with 5% NH_4_OH in 30% methanol.

After vacuum-dried, the iTRAQ-labeled peptides were reconstituted with 20 μl of 5 mM KH_2_PO_4_ containing 5% acetonitrile (pH 3.0), and separated via 2D-Liquid Chromatography (2D-LC) with an Ultimate Dual-gradient LC system (Dionex Ultimate3000).

### Analysis of liquid chromatography mass spectrometry (LC-MS)

The LC-MS analysis was performed on a Triple-TOF 5600 system (AB SCIEX). Mass spectra were collected (m/z 400–1250) in high resolution (> 30,000), using 250 ms per spectrum. A maximum of 50 precursors in a cycle were selected for fragmentation from each mass spectrum. Tandem mass spectra were harvested in high sensitivity mode (resolution > 15,000).

The analyses of peptide and protein identifications and iTRAQ quantification were performed on GPS Explorer software (Applied Biosystems) using MASCOT search engine (Matrix Science). The NCBI database (http://www.ncbi.nlm.nih.gov/protein/?term=txid9615[Organism:noexp]) was selected for analysis. Cysteine methane thiolation, iTRAQ-labeled lysine, and N-terminal iTRAQ labeling were selected as fixed modifications in the data analysis (Zieske, 2006).

Two-sample unequal variant-test was selected to analyze the difference between mock-infected and infected samples. The different proteins were corresponded to a *P*-value < 0.05. Threshold for iTRAQ was defined according to 95% CV+1.

### Protein pathway analysis

Data were analyzed with IPA (Ingenuity Systems, http://www.ingenuity.com), and proteins networks were constructed as previously introduced (Munday et al., 2010). Briefly, differentially identified proteins were uploaded into the IPA and then corresponding genes were mapped using the Ingenuity Pathways Knowledge Base. The connections between the genes were further examined basing on the information of the database. Graphical networks for these genes were constructed basing on their connectivity algorithms. The nodes represented genes or gene products. The color intensity of the node indicates the relative decrease (green) or increase (red) abundance. Different shapes of the nodes represent the functional class of the gene. The nodes were linked based on the biological relationships.

### Real-time RT-PCR

For the differently expressed proteins identified, selected proteins with known sequence and function were validated by real-time PCR. Lung tissue samples were homogenated in ice-cold PBS. Total tissue RNA was extracted using the RNeasy Mini Kit (QIAGEN) following the manufacturers' protocol. RNA concentrations were measured by a spectrophotometer (260 nm/280 nm), followed by generation of cDNA with Reverse-transcription Kit (Invitrogen). Quantitative real-time PCR was performed to determine the expression of GAPDH (glyceraldehyde-3-phosphate dehydrogenase) gene and the targeted genes with TaqMan Universal PCR Master Mix kit (Applied Biosystems). After initial denaturation at 95°C for 30 s, amplification was conducted for 40 cycles with denaturation at 95°C for 15 s, primer annealing at 53°C for 30 s, and DNA extension at 72°C for 15 s. Primers used were: Annexin A2 forward: 5′-CTATGATGCTGGAGTGAAGAG-3′ and reverse: 5′-GGAAAGCATTTTCCAGGTCTC-3′; Caspase9 forward: 5′-ACATTGGTTCTGGCGGAGCTC-3′ and reverse: 5′- TCACGGTCCAAGTTGGAGCCC-3′; Mx1 forward: 5′-TGATATGCTGCACACGATAAC-3′ and reverse: 5′-GATCTGCTCCATTTGGAAGTG-3′; Bcl-xl forward: 5′-TGACATCCCAGCTTCACATCA-3′ and reverse: 5′-CTGCATCTCCTTGTCTACGCT-3′; GAPDH forward: 5′-CATCACCATCTTCCAGGAGCG-3′ and reverse: 5′-AGATGATGACCCTTTTGGCT-3′.

### Western blot analysis

For western blot analysis, total proteins from lung tissue samples were extracted, and the protein concentration was determined using the Bradford assay. Cell lysate (30 μg) were fractionated by 12% SDS-PAGE, followed by transferring to membranes (GE Healthcare). After blocking with 5% non-fat dried milk, the membranes were incubated, respectively with rabbit/mouse monoclonal or polyclonal antibodies to GAPDH (ab9484), Annexin A2 (ab41803), Caspase-9 (ab63488), Mx1 (ab95926), and Bcl-xl (ab32670) (Abcam). The membranes were then separately incubated with horseradish peroxidase (HRP)-conjugated goat anti-mouse/rabbit IgG (used at 1:1000 dilutions, Santa Cruz Biotechnology). The secondary antibody was detected with enhanced chemiluminescence reagent (Thermo Scientific).

### Statistical analysis

Statistical significance of differences between groups was determined by using a paired, non-parametric Student's *t*-test. *P* < 0.05 is considered as statistically significant.

## Results

### Experimental infection with canine H3N2 influenza virus in dogs

**Figure 1:**
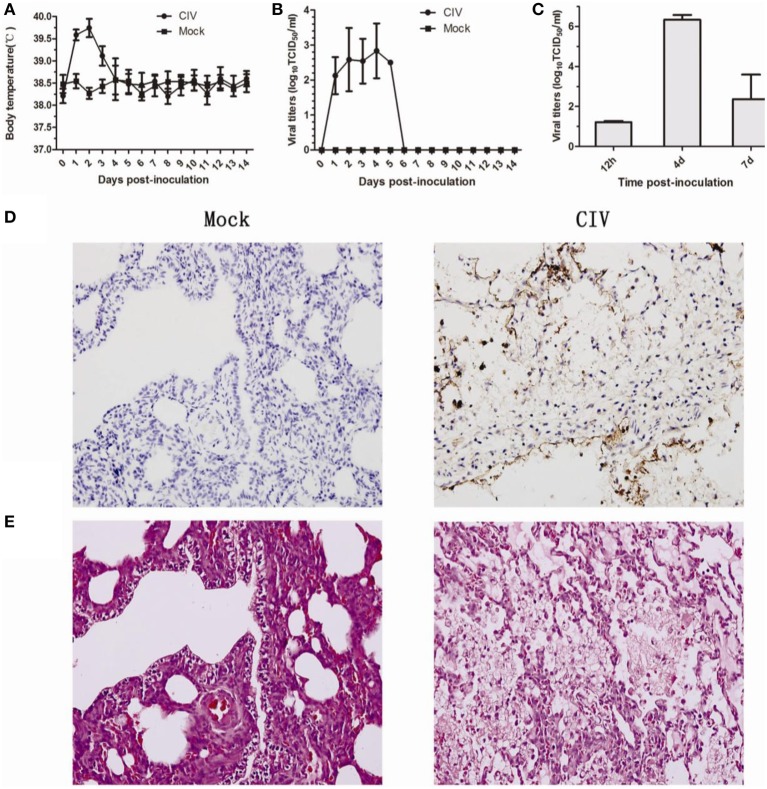
**Pathology of dogs experimental infected with H3N2 canine influenza virus**. Dogs were infected with 10^6^ EID_50_ of virus by intranasal inoculation. Rectal body temperature **(A)** and, the viral titers **(B)** of nasal swabs were monitored for 14 days, and viral replication in the lungs at 12 hpi, 4 dpi, and 7 dpi **(C)**. Lung tissues from infected or mock-infected dogs were stained with rabbit anti-influenza A nucleoprotein (NP) antibody, HRP-labeled goat anti-rabbit secondary antibody, and red alkaline phosphatase substrate **(D)**. Lung tissues from infected or mock-infected dogs were stained with H&E stain **(E)**.

All dogs inoculated with the CIV showed running nose, coughs, and elevated body temperatures. The temperature peaked at 40.1°C at 2 dpi (Figure 1A). Nasal swabs were collected from 1 to 14 dpi, viruses were detected from all infected dogs from 1 to 5 dpi, and viral titers peaked at 4 dpi at 10^2.85^ TCID_50_ (Figure 1B). The viral replication of lung tissues were detected at 12 hpi, 4 dpi, and 7 dpi, and the mean viral titers of 12 hpi, 4 dpi, and 7 dpi were 1.21, 6.33, and 2.37 logTCID50/ml, respectively (Figure 1C). Seroconversions were detected at 14 dpi (not shown). For the gross pathology examinations, lesions were found in lungs of infected dogs which were characterized by severe reddish consolidation, especially involving the anterior lobes (not shown). Additionally, the lung tissues stained with the antibody which against influenza A NP protein (obtained from Sigma-Aldrich) was positive mainly in pneumocytes (Figure 1D). The H&E staining of the lung tissues showed interstitial pneumonia (Figure 1E). These results showed that avian-origin CIV H3N2 could cause respiratory diseases in the dogs experimentally inoculated with the virus.

### Identification of differentially expressed lung proteins

**Figure 2:**
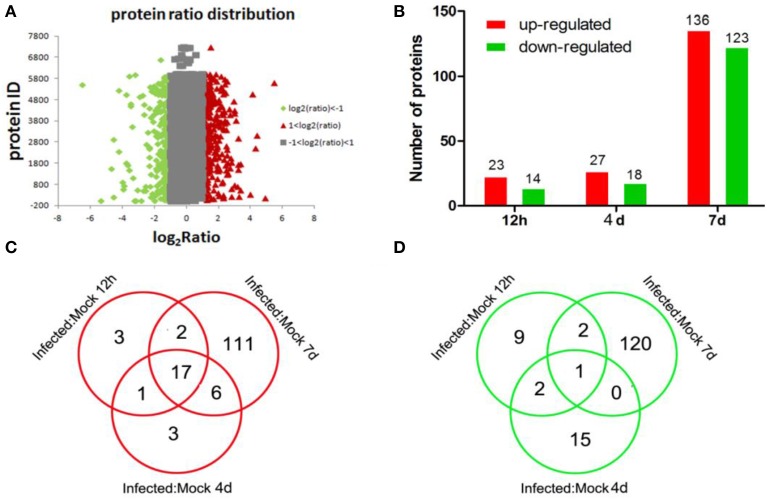
**Distributions of total, up-regulated, and down-regulated differential expressed proteins**. Distributions of total proteins ratio **(A)**, analysis of up-regulated and down-regulated proteins identified at different time points **(B)**; distributions of up-regulated **(C)** and down-regulated **(D)** proteins identified at different time points as indicated.

To identify differentially expressed proteins, animals were euthanized at 12 hpi, 4 dpi, and 7 dpi, and their lung tissue samples were collected for iTRAQ analysis. Relative protein expression values were compared between infected and mock-infected animals. A total of 17,796 proteins were detected and quantified. The ratio of expression level of GAPDH, a reference control, was at equivalent between the levels from infected and mock-infected dog samples. We further filtered statistically significant proteins with the adjusted *P*-value < 0.05 (95% confidence) (Figure 2A). Of these, 23, 27, and 136 proteins were highly up-regulated with ratios ≥2 at 12 hpi, 4 dpi, and 7 dpi, respectively (Table S1) (Figure 2B), and 14, 18 and 123 were significantly down-regulated with ratios ≤0.5 (Table S1) (Figure 2B). As the infection progressed, the number of differentially expressed proteins increased (Table S1) (Figure 2B). Among the up-regulated proteins, 17 proteins were presented at all three different time points (Table 1) (Figure 2). These proteins included anti-viral proteins (OAS1-3, TRIM22, Mx1-2, IFGGB1-2, ISG15, ISG20 and, pulmonary surfactant-associated protein A-B) and proteins involved in antigen presentation and signal transduction. The higher expression levels of these IFN response-related genes were detected at 12 hpi and 4 dpi. At day 7, the expression of these genes was lowered. Among the down-regulated proteins, Calcyphosin was presented at all three different time points (Table 1) (Figure 2D). Calcyphosin is a calcium-binding protein, which may play a role in the regulation of ion transport and may be associated with interstitial lung disease. The MX (Horisberger et al., 1983; Kolb et al., 1992; Haller et al., 1998), TRIM22 (Di Pietro et al., 2013), ISG15 (Hsiang et al., 2009), OAS (Hale et al., 2008), and SFTPA1, B (Reading et al., 1997; Hartshorn et al., 2000) gene are important effectors of innate immune response against viral infections. Up-regulation of IFN inducible proteins WARS and TAP1 (Coombs et al., 2010; Wu et al., 2013) has been reported previously, but their roles during influenza virus infection remain to be clarified.

**Table 1:**
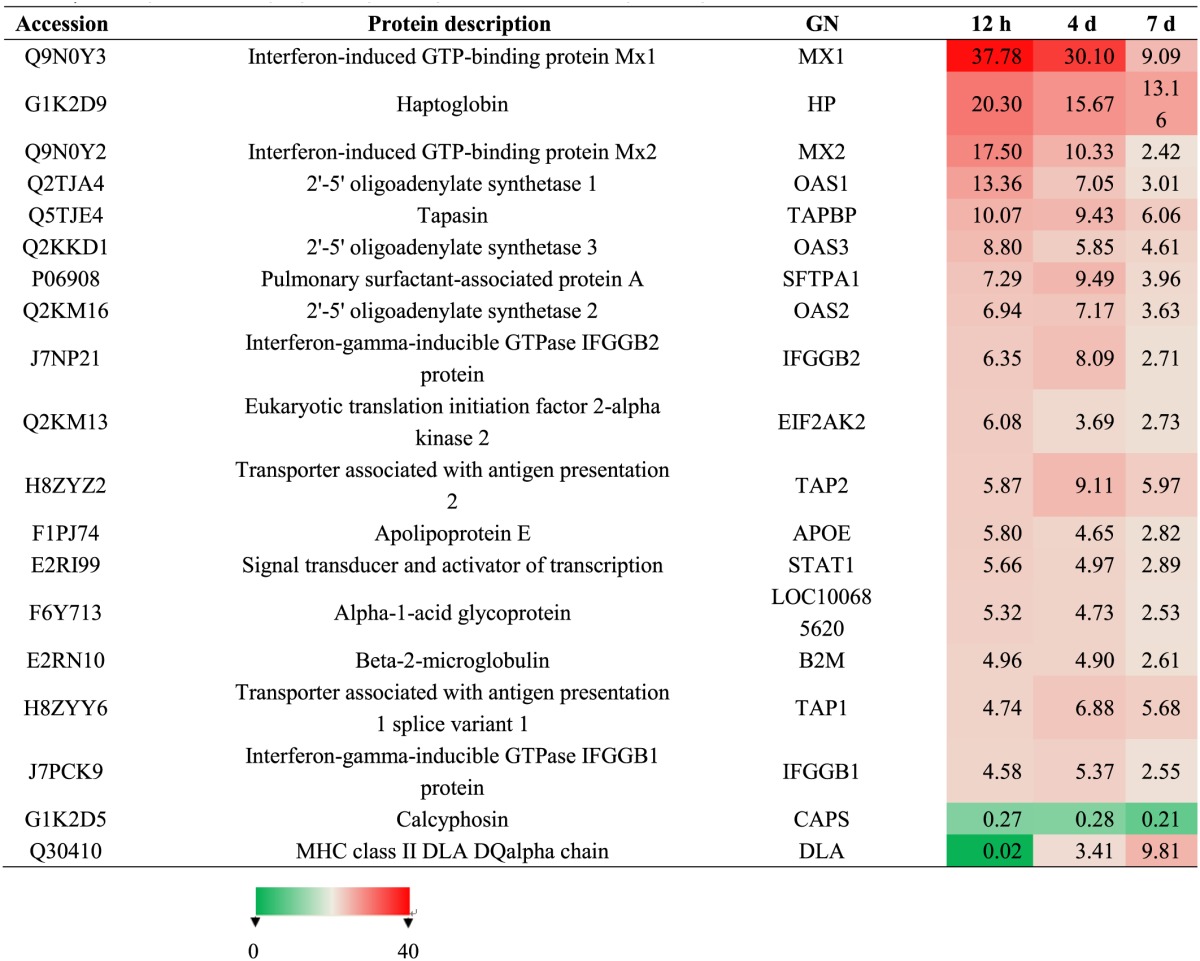
**Summary of differentially expressed proteins presented at hour 12, day 4 and 7 post-inoculation**.

### Subcellular and functional characterization of identified proteins and bioinformatics analysis

**Figure 3:**
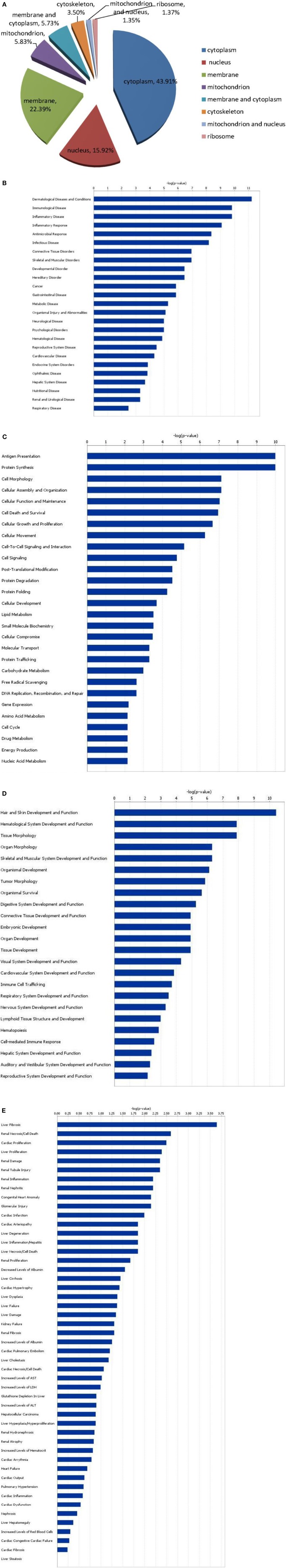
**Subcellular localization of differentially expressed proteins (A), and functional characterization of up-regulated and down-regulated proteins (B–E). (B)** Diseases and disorders; **(C)** molecular and cellular functions; **(D)** physiological system development; **(E)** functions and toxicity functions.

To gain functional insights into the cellular proteome, these identified proteins were assigned to different molecular function and subcellular annotations based on the biology evidence from the UniProt KB/Swiss-Prot and TrEMBL protein database and from the Gene Ontology database. We examined the subcellular localizations of the 278 identified proteins. These proteins (Figure 3A) were localized in the cytoplasm (43.91%), nucleus (15.92%), membrane (22.39%), mitochondrion (5.83%), membrane and cytoplasm (5.73%), cytoskeleton (3.5%), mitochondrion and nucleus (1.35%) and ribosome (1.37%).

Grouping of the previously characterized 278 proteins revealed that their functions differed between up-regulated and down-regulated proteins. Molecular and cellular functions, diseases and disorders, physiological system development and functions, and toxicity functions that were determined with statistically significant levels (*p* = 0.05) are depicted in Figures 3B–E. The 278 differentially expressed proteins correspond to 25 diseases and disorders (Figure 3B), including dermatological diseases and conditions, immunological diseases, inflammatory diseases, inflammatory response, antimicrobial response, and infectious diseases. These proteins can also be assigned to 28 molecular and cellular functional groups (Figure 3C), including antigen presentation, protein synthesis, cellular morphology, cellular assembly and organization, and cell death; 24 physiological system development and functional groups (Figure 3D), including hair and skin development and function, hematological system development and function, tissue morphology, organ morphology, and skeletal and muscular system development and function; and 49 toxicity functions groups (Figure 3E), including liver hepatitis, renal necrosis/cell death, cardiac proliferation, liver proliferation, and renal damage.

**Figure 4:**
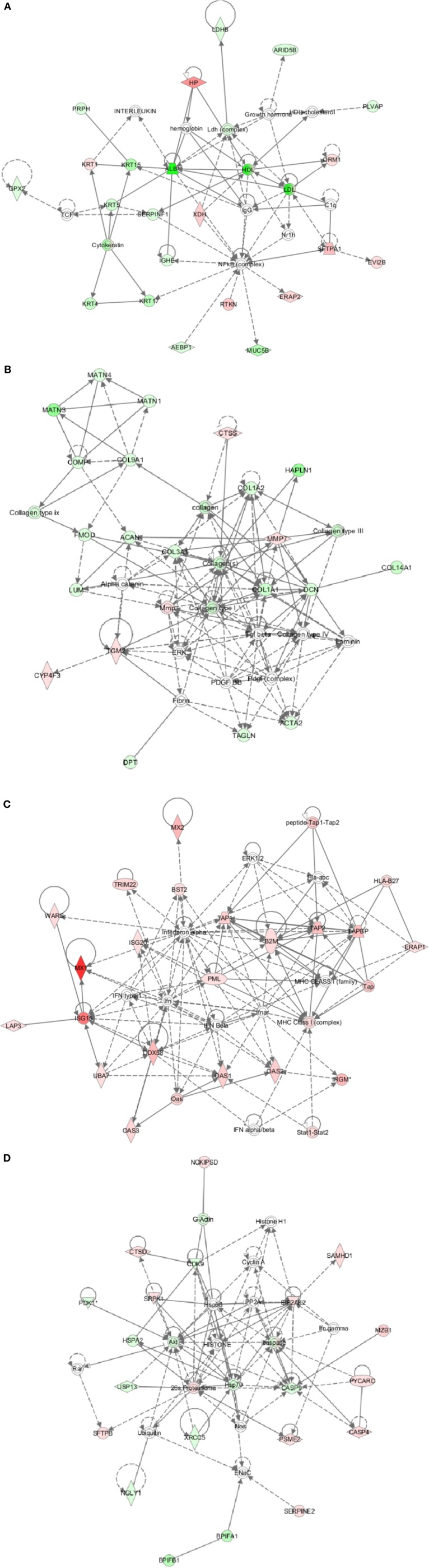
**Ingenuity pathway analysis (IPA) of differentially expressed proteins. (A)** Cancer, free radical, scavenging, dermatological disease, and conditions; **(B)** Cancer, developmental disorder, and muscular and skeletal disorders; **(C)** Antimicrobial response, inflammatory diseases, and antigen presentation; **(D)** Hematological disease, immunological diseases, and infectious disease. Red, significantly up-regulated proteins; pink, moderately up-regulated proteins; light green, moderately down-regulated proteins; dark green, significantly down-regulated proteins; white, proteins known to be in the network but not identified in our study.

Different proteins were defined with four specific functional networks and each network contained at least eight members. The four networks of interest correspond to: (1) cancer, free radical, scavenging, dermatological disease and conditions (Figure 4A); (2) cancer, developmental disorder, and muscular and skeletal disorders (Figure 4B); (3) antimicrobial response, inflammatory diseases and antigen presentation (Figure 4C); (4) hematological disease, immunological diseases and infectious disease (Figure 4D).

### Validation of transcriptional profiles and western blot of differentially expressed proteins

**Figure 5:**
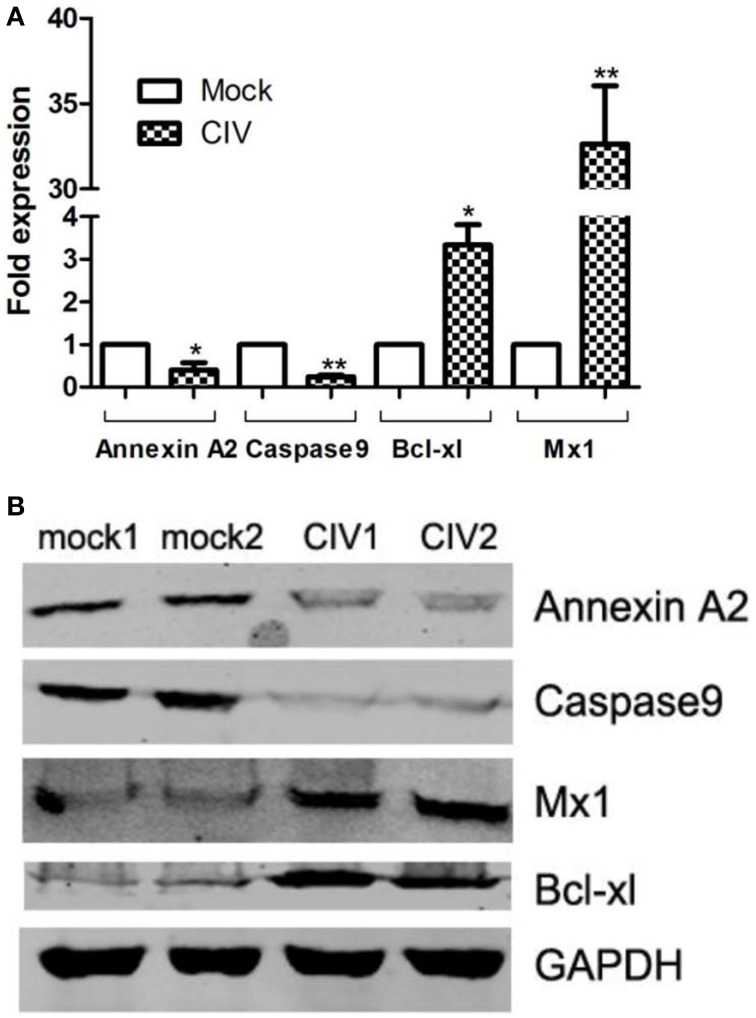
**Transcriptional modulation and Western blot confirmation of representative proteins**. CIV-infected canine lung tissues at 4 dpi are harvested. **(A)** Total RNAs from infected/uninfected lung tissues were measured by real-time RT-PCR analysis. Samples were normalized with the GAPDH gene as the control housekeeping gene. **(B)** Expression of Annexin A2, Caspase9, Bcl-xL, and Mx1 in CIV-infected canine lung tissues is indicated by the Western blot. Expression of GAPDH in from different lung samples was evaluated as a normalization method. Consistent results were obtained by three independent experiments.

To confirm the protein quantification, four proteins with altered abundances (Annexin A2, Caspase9, Mx1, and Bcl-xL) were chosen to validate the results. The transcriptional alterations of these four selected genes in the infected lung tissues were analyzed. As shown in Figure 5A, the trends in the change of the four genes' mRNA levels in the infected and uninfected lung tissues were similar to the changed patterns as what was observed in iTRAQ analysis. To confirm the protein expression level of these four genes of Annexin A2, Caspase-9, Mx1, and Bcl-xL during infection, equal amounts proteins were examined by Western blot with corresponding specific antibodies (Figure 5B). The result showed that the expression levels of Annexin A2 and Caspase9 in infected dogs were lower than that in controls, and the expression of Mx1 and Bcl-xL was higher than that in controls. These data agreed with the results obtained in iTRAQ analysis.

## Discussion

Interactions between virus and host during an infection involve a complex interplay of host cellular and viral networks. To evade host resistance and immunity, and to adapt to the new host, the virus modulates various host cellular processes to increase viral replication efficiency. On the other hand, for the host to achieve anti-viral state, specific cellular processes are either activated or suppressed. These changes are reflected by the differential expression of cellular proteins. High-throughput quantitative proteomics using iTRAQ is an ideal method to map such changes. In addition, quantitation of differentially expressed proteins by this technique is independent of the number of cells. Therefore, it is well suited for studies using animal tissues or multi-cell samples. However, as lung tissues usually contain heterogeneous cell types and the protein expressions in different cells and locations may vary, the proteomic analysis in this study can only reflect the average level of gene expressions at the selected lobus cardiacus in the right lungs of the inoculated dogs. Whole genomic screening using single cell based techniques may help to elucidate the differential expression of proteins in the different pulmonary cells.

The time of 12 hpi was used to represent early infection when the innate immune molecules began to identify the virus infection and launch the anti-viral response. At this time point, we can find which innate immune molecules are involved. The time of 4 dpi was the peak period for virus replication in the lungs. The peak of viral replication may be due to the incomplete clearance by innate immunity at early infection. Key anti-viral proteins may be revealed at this time. Specific immunity would be started at around 7 dpi to clear the virus. It was observed that at 12 hpi and 4 dpi, the number and types of differently expressed peptides were less than that at 7 dpi. This reflected the rapid and robust inflammatory response induced at the early phase of the infection, and the decrease of relative level of most immune molecules at 7 dpi. In addition, the most differentially expressed proteins including signal transduction molecules, kinases and other biochemical metabolism related enzymes, appeared at 7 dpi, and may be associated with the repair of damaged lung tissues.

In this study, by characterizing the proteomics of infected canine lungs in response to avian-origin CIV (H3N2), we have identified cellular processes, such as gene expression, signaling pathways and morphology altered by viral infection. A total of 278 differentially expressed proteins were identified, including cellular proteins involved in cellular growth, proliferation, assembly and organization, and apoptosis.

These differentially expressed proteins correspond to 25 diseases and disorders such as dermatological conditions, immunological diseases, inflammation, antimicrobial response, and infectious diseases. Twenty-two proteins were involved in dermatological diseases and conditions, which were identified as most active during diseases and immune disorders models. Interestingly, these differentially expressed proteins were not directly involved in respiratory diseases or conditions. One implication is that the clinical signs, such as fever and pneumonia in inoculated dogs were not a direct result of virus infection, but were caused by the immune response, parallel to the “cytokine storm” in severe clinical diseases (Julkunen et al., 2001; Tisoncik et al., 2012), particularly in HPAI H5N1 infections (Seo et al., 2002).

Interferon (IFN) produced in response to pathogens, is an extremely powerful component of the host innate immunity and works as the first defense line against viral infection/replication. Activation of IFN signaling pathways results in the production of interferon-stimulated genes (ISGs), many of which have antiviral activities. Indeed, many interferon induced proteins were up-regulated after infection. Among these, the expression of Mx1, Mx2, TRIM22, WARS, ISG15, ISG20, UBA7, DDX58, and OAS1-3 were up-regulated, representing an increased innate immune response against viral infections. The higher expression levels of these interferon response-related genes were detected at 12 hpi and 4 dpi, which may be associated with CIV infection-induced inflammation. At 7 dpi the expression of these genes was lowered, which may be associated with the inhibited replication of virus by the host response.

A previous report has showed that these proteins in monkey lungs involved in the innate immune response were up-regulated after infection with influenza A virus [A/Texas/36/1991(H1N1)] (Baas et al., 2006), and the trends of the protein expression were consistent with those observed in our study. However, similar results were not found in other proteomics research on mice lungs infected with H5N1 (Zhao et al., 2012) and H9N2 (Qi et al., 2015) AIV, and chicken brain infected with HPAI H5N1 (Zou et al., 2010). The cellular proteomics data obtained from A549 cells infected with H3N2 swine influenza virus and H1N1 human influenza virus showed that Mx1, Mx2, and ISG15 were up-regulated and some cytoskeleton proteins were down-regulated (Coombs et al., 2010; Dove et al., 2012; Wu et al., 2013).

Mx1 can prevent the vRNPs virus from being transported to the nucleus (Xiao et al., 2013) as well as facilitating a direct interaction with the between MxA and influenza A virus NP protein (Zimmermann et al., 2011). Mx2 protein inhibits hantavirus (Jin et al., 2001) and HIV-1 (Goujon et al., 2013) but not influenza virus replication. TRIM22 can inhibit RNA viruses infection such as encephalomyocarditis virus (ECMV), hepatitis B virus (HBV), human immunodeficiency virus type 1 (HIV-1) and influenza virus (Di Pietro et al., 2013). IFN-induced antiviral activity against influenza virus is by reducing protein synthesis by 5 to 20-fold. This action is implemented by suppressing ISG15 conjugation (Hsiang et al., 2009). The genes WARS, OAS1-3, ISG20, UBA7, and DDX58 (Figure 4C) showed a strong response to CIV (H3N2) infection, but the effect on viral replication remains to be determined. In addition, the pathway for antigen presentation mediated by MHC class I was strongly induced by CIV (H3N2) infection (Figure 4C). Many genes related to cellular immunity were also up-regulated; e.g., beta-2-microglobulin (β2m), TAP1, TAP2, TAPBP, and IRGM. The function of β2m is mainly in the stabilization of the tertiary structure of the MHC class I to permit the presentation of antigenic peptides to CD8+ cytotoxic T lymphocytes (Yu et al., 2013). Transporter associated with antigen presenting (TAP) 1 and TAP2 genes are localized in the major histocompatibility complex (MHC) class II region and can form a heterodimer. These proteins play a key role in endogenous pathways for antigen presentation. Expression of HLA-DQA1 was also up-regulated. Overall, these results show that CIV (H3N2) infection induces a strong immune response.

Other differentially expressed proteins identified, such as surfactant protein A1 (SFTPA) and surfactant protein B (SFTPB), were also up-regulated in our study. SFTPA and SFTPB can bind to the polysaccharides of microorganisms and are considered as an important function in innate immunity (Reading et al., 1997). SFTPA and SFTPB can inhibit influenza viral infectivity by binding to the viral HA or NA (Benne et al., 1997; Hartshorn et al., 1997, 2000; Reading et al., 1997). Previously, Qi et al. has demonstrated that the increased pathogenicity for LPAI H9N2 PB2 627K may be associated with SFTPA (Qi et al., 2015), but in monkey lungs infected with H1N1, it was found that surfactant protein D (SFTPD) was down-regulated (Baas et al., 2006). In general, CIV (H3N2) infection activates both innate and adaptive immune response and the virus is eliminated quickly from the host.

Infection of influenza virus in the respiratory tract causes cell damage and induction of cytokines and chemokines. These molecules trigger inflammatory and respiratory distress symptoms. Cell death is by apoptosis, which is characterized by specific morphological changes, including disruption of cytoskeleton, condensation of the cytoplasm and chromatin, membrane blebbing, loss of mitochondrial function, and fragmentation of DNA. As shown in Figure 4B, a variety of cytoskeleton-associated proteins, such as actin, collagen, matrilin were down-regulated upon infection with the CIV in dogs. Notably, HPAI H5N1 infection in chicken caused the up-regulation of brain cytoskeleton (Zou et al., 2010); H9N2 AIV infection in mice led to the decrease of lung cytoskeleton proteins including moesin, ezrin, and myosin et al (Qi et al., 2015).

Two other proteins associated with cell death and apoptosis, B-cell lymphoma-extra-large (Bcl-xL) and Caspase-9, were identified as being up-regulated and down-regulated by CIV (H3N2) infection. Bcl-xL is a member of the Bcl-2 protein family whose main function is to promote cell survival; Caspase-9 can catalyze Caspase-3, Caspase-6, and Caspase-7. Caspase-7 can activate the caspase cascade and promotes apoptosis. Influenza virus-induced apoptosis may be a host defense mechanism (Xie et al., 2009). However, lung tissue injury may also be associated with apoptosis. Therefore, induction of apoptosis is a host defense mechanism by which the replication and spread of virus is blocked. Inhibiting influenza virus-induced apoptosis by Bcl-2 expression reduces virus yield, virus spread, and glycosylation of viral protein such as, the HA (Saito et al., 1996; Nencioni et al., 2009). Inhibition of apoptosis happened during CIV infection could limit virus replication and reduce the lung damage. Compared to results obtained from the influenza virus-infected A549 cells (Kroeker et al., 2012), it was showed that apoptosis associated-proteins were down-regulated in both studies, though the proteins related to cell death signaling were not exactly the same.

Although complete genome of dogs has been sequenced (Lindblad-Toh et al., 2005), full annotation remains to be completed. This study is a first attempt to combine high-throughput analysis and the canine genome data to understand the molecular basis of a newly emerged virus in the new host. Despite limitation of known functions for many differentially expressed proteins, our results provide informaion for more refined understanding of interspecies transmission, viral pathogenesis, and host response.

## Author contributions

SS, JT, MH, and PZ conducted the experiments; analyzed the data and wrote the paper. GL participated in animal experiment. HZ, AL, and GZ provided technical support. SS, JT, HZ, AL, and SL desired the experiments and revised paper.

### Conflict of interest statement

The authors declare that the research was conducted in the absence of any commercial or financial relationships that could be construed as a potential conflict of interest.
