# Supplementary material for: Global and quantitative proteomic analysis of dogs infected by avian-like H3N2 canine influenza virus
Source: Front Microbiol. 2015 Apr 2;6:228. doi: 10.3389/fmicb.2015.00228 (PMC4382988; doi:10.3389/fmicb.2015.00228)
Supplement: Supplementary file 1 [file Table1.DOCX]

**Table S1.** Summary of Proteins Differentially Expressed in Lung Tissues at hour 12, day 4 and 7 post-inoculation.

| Accession | Protein description | GN | **12 h** | **3 d** | **7 d** |
| --- | --- | --- | --- | --- | --- |
|  |  |  |  |  |  |
| E2R434 | Palmitoyltransferase ZDHHC5 | ZDHHC5 | **0.77** | **0.89** | **0.24** |
| F1PQ83 | Palmitoyltransferase | ZDHHC18 | **0.53** | **1.00** | **0.49** |
| J9P1Y9 | Protein yippee-like | YPEL5 | **0.19** | **0.22** | **3.07** |
| F1PSB7 | Tyrosine-protein kinase Yes | YES1 | **0.90** | **1.00** | **2.03** |
| Q9TSX8 | Pantetheinase | VNN1 | **1.01** | **3.91** | **7.51** |
| F1PAX0 | Pantetheinase | VNN1 | **1.43** | **0.41** | **0.40** |
| P50551 | Vasodilator-stimulated phosphoprotein | VASP | **0.79** | **7.08** | **5.98** |
| F1PRR4 | Vasodilator-stimulated phosphoprotein | VASP | **0.89** | **0.88** | **2.03** |
| J9P005 | Ubiquitin carboxyl-terminal hydrolase | USP35 | **0.94** | **1.08** | **2.99** |
| E2R7B0 | Ubiquitin carboxyl-terminal hydrolase | USP13 | **0.86** | **2.73** | **0.25** |
| E2R311 | UDP-glucose 6-dehydrogenase | UGDH | **0.29** | **0.21** | **1.13** |
| Q9GL21 | Uveal autoantigen with coiled-coil domains and ankyrin repeats | UACA | **0.33** | **3.23** | **0.08** |
| Q9XSB8 | Tripeptidyl-peptidase 1 | TPP1 | **0.39** | **21.00** | **12.45** |
| Q8HX86 | Thiopurine S-methyltransferase | TPMT | **0.67** | **0.24** | **0.61** |
| F1PRA7 | DNA topoisomerase 2 | TOP2B | **0.53** | **0.88** | **2.01** |
| J9P749 | Tight junction protein ZO-2 | TJP2 | **0.86** | **0.11** | **3.29** |
| F1PMA1 | Tight junction protein ZO-1 | TJP1 | **0.95** | **0.96** | **0.47** |
| P43698 | Thyroid transcription factor 1 | TITF1 | **0.88** | **0.51** | **5.66** |
| F1PB37 | Thrombomodulin | THBD | **0.50** | **1.03** | **0.40** |
| A0M8U6 | Testin | TES | **0.82** | **0.58** | **0.09** |
| J9NXE6 | Tektin-1 | TEKT1 | **1.04** | **1.30** | **2.75** |
| Q863A2 | T-box transcription factor TBX2 | TBX2 | **0.98** | **0.19** | **0.70** |
| Q5TJE4 | Tapasin | TAPBP | **10.07** | **9.43** | **6.06** |
| H8ZYZ2 | Transporter associated with antigen presentation 2 | TAP2 | **5.87** | **9.11** | **5.97** |
| H8ZYY6 | Transporter associated with antigen presentation 1 splice variant 1 | TAP1 | **4.74** | **6.88** | **5.68** |
| F1PSC8 | Tyrosine-protein kinase | SYK | **0.29** | **0.72** | **2.03** |
| Q29476 | Sulfotransferase 1A1 | SULT1A1 | **0.55** | **0.54** | **11.01** |
| Q28288 | Syntaxin-binding protein 2 | STXBP2 | **1.02** | **1.01** | **9.67** |
| B6F250 | Erythrocyte band 7 integral membrane protein stomatin | STOM | **0.78** | **0.85** | **0.14** |
| J9NVP2 | Stathmin | STMN1 | **0.92** | **0.71** | **2.71** |
| E2RI99 | Signal transducer and activator of transcription | STAT1 | **5.66** | **4.97** | **2.89** |
| F1PLH3 | Signal recognition particle receptor subunit alpha | SRPR | **0.69** | **0.98** | **0.47** |
| Q2KNA0 | Cytospin-A | SPECC1L | **0.76** | **1.22** | **12.00** |
| F1PAS8 | Superoxide dismutase [Cu-Zn] | SOD3 | **0.64** | **0.62** | **0.39** |
| E2RSF2 | Superoxide dismutase | SOD2 | **1.10** | **1.04** | **0.35** |
| F1PUL0 | O-acyltransferase | SOAT1 | **1.19** | **1.75** | **2.09** |
| E2RCX4 | Structural maintenance of chromosomes protein | SMC4 | **0.79** | **0.92** | **0.25** |
| F1PXA1 | Transporter | SLC6A4 | **0.52** | **0.93** | **2.13** |
| Q2Z1P8 | Solute carrier family 4, anion exchanger, member 1 (Erythrocyte membrane protein band 3) | SLC4A1 | **0.78** | **0.85** | **14.49** |
| F1PPK3 | Solute carrier family 2, facilitated glucose transporter member 3 | SLC2A3 | **0.42** | **0.82** | **0.49** |
| K7ZSN9 | Solute carrier family 1 (Neutral amino acid transporter), member 5 | SLC1A5 | **0.81** | **0.72** | **3.40** |
| E2R3C6 | SH3 domain-binding glutamic acid-rich-like protein | SH3BGRL2 | **0.32** | **0.98** | **0.23** |
| E2RH95 | SH3 domain-binding glutamic acid-rich-like protein | SH3BGRL | **0.62** | **1.18** | **0.32** |
| F1PGB5 | Pulmonary surfactant-associated protein C | SFTPC | **0.79** | **0.77** | **0.44** |
| P17129 | Pulmonary surfactant-associated protein B | SFTPB | **0.82** | **1.00** | **4.73** |
| P06908 | Pulmonary surfactant-associated protein A | SFTPA1 | **7.29** | **9.49** | **3.96** |
| F1Q1W7 | Pulmonary surfactant-associated protein A | SFTPA1 | **0.60** | **0.81** | **2.31** |
| P13679 | Signal peptidase complex catalytic subunit SEC11C | SEC11C | **0.82** | **0.93** | **4.70** |
| E2RQU4 | Ribosomal protein S6 kinase | RPS6KA1 | **0.49** | **0.49** | **0.34** |
| F2Z4N3 | 60S ribosomal protein L27 | RPL27 | **0.13** | **0.88** | **2.42** |
| E2RL34 | 60S ribosomal protein L18a | RPL18A | **0.23** | **0.83** | **0.33** |
| D0VWQ3 | Ribosomal protein L18 | RPL18 | **0.73** | **0.80** | **0.15** |
| E2QXF3 | 60S ribosomal protein L15 | RPL15 | **0.69** | **1.12** | **0.18** |
| J9JHS0 | Tyrosine-protein kinase transmembrane receptor | ROR2 | **0.71** | **0.39** | **2.54** |
| F1PP14 | Mitochondrial Rho GTPase | RHOT1 | **1.79** | **0.69** | **0.48** |
| Q00M95 | Inactive rhomboid protein 2 | RHBDF2 | **1.12** | **0.75** | **7.41** |
| P18067 | Ras-related protein Rab-7a | RAB7A | **0.50** | **1.03** | **4.82** |
| Q1HE58 | Ras-related protein Rab-27A | RAB27A | **0.87** | **0.96** | **8.91** |
| P51152 | Ras-related protein Rab-12 | RAB12 | **0.65** | **0.94** | **6.09** |
| J9NZ55 | Nicotinate-nucleotide pyrophosphorylase [carboxylating] | QPRT | **5.22** | **1.92** | **2.78** |
| F1PB77 | Phosphorylase | PYGL | **0.70** | **0.81** | **0.40** |
| F1PSM2 | Phosphorylase | PYGB | **0.37** | **0.56** | **2.05** |
| E2RFR8 | Tyrosine-protein phosphatase non-receptor type | PTPN6 | **0.72** | **0.39** | **0.29** |
| F1PLF3 | Tyrosine-protein phosphatase non-receptor type | PTPN2 | **0.74** | **0.46** | **0.47** |
| F1Q1Z8 | Tyrosine-protein phosphatase non-receptor type | PTPN1 | **0.59** | **1.34** | **2.31** |
| Q5W412 | Proteasome subunit beta type | PSMB9 | **0.89** | **1.03** | **1.31** |
| Q5W416 | Proteasome subunit beta type-8 | PSMB8 | **1.17** | **0.93** | **0.11** |
| E2RKR9 | Proteasome subunit beta type | PSMB10 | **0.57** | **2.27** | **0.32** |
| E2RF52 | Proteasome subunit alpha type | PSMA8 | **0.61** | **1.02** | **0.28** |
| E6DEI5 | Precorrin-3B C(17)-methyltransferase | Propioni | **0.32** | **0.51** | **0.36** |
| E2RNP2 | Protein kinase C | PRKCQ | **1.13** | **0.86** | **0.33** |
| F1PDX2 | Protein kinase C delta type | PRKCD | **0.74** | **1.05** | **0.44** |
| F1PKU7 | Protein kinase C | PRKCB | **3.69** | **1.05** | **0.45** |
| F1Q1D5 | Protein kinase C | PRKCA | **0.86** | **0.77** | **2.28** |
| Q5JZR0 | Palmitoyl-protein thioesterase 1 | ppt1 | **0.92** | **0.91** | **1.05** |
| F1PV61 | Serine/threonine-protein phosphatase | PPP5C | **0.89** | **0.95** | **2.12** |
| E2R7N5 | Coronin | PPP1CA | **1.08** | **3.27** | **0.25** |
| J9NUZ6 | Peptidyl-prolyl cis-trans isomerase | PPIC | **0.88** | **0.04** | **2.61** |
| E2QYJ4 | DNA-directed RNA polymerase subunit | POLR2I | **0.65** | **0.83** | **0.19** |
| E2RP20 | Perilipin | PLIN2 | **0.78** | **0.98** | **0.34** |
| F1Q421 | Plasminogen | PLG | **0.66** | **2.39** | **2.39** |
| Q6Q308 | Pleckstrin | PLEK | **0.82** | **0.02** | **0.17** |
| F1P9E5 | Platelet-activating factor acetylhydrolase | PLA2G7 | **0.65** | **0.72** | **0.38** |
| F1PHR2 | Pyruvate kinase | PKM | **0.93** | **0.77** | **0.45** |
| E2RRC9 | Phosphoglycerate kinase | PGK1 | **0.70** | **0.14** | **0.35** |
| F1PE09 | 6-phosphogluconate dehydrogenase, decarboxylating | PGD | **0.73** | **0.88** | **0.44** |
| F1PKS1 | 6-phosphofructokinase | PFKP | **0.47** | **1.18** | **0.45** |
| P52784 | 6-phosphofructokinase, muscle type | PFKM | **0.80** | **1.34** | **6.69** |
| Q6QNF3 | Platelet-derived growth factor receptor beta | PDGFRB | **0.78** | **0.04** | **0.16** |
| O77746 | cGMP-specific 3',5'-cyclic phosphodiesterase | PDE5A | **0.76** | **0.43** | **3.67** |
| F1PJ13 | Succinyl-CoA:3-ketoacid-coenzyme A transferase | OXCT1 | **0.72** | **0.79** | **0.45** |
| J9PB04 | Oxysterol-binding protein | OSBPL2 | **0.75** | **0.84** | **3.40** |
| F1PNG2 | Oxysterol-binding protein | OSBPL11 | **0.74** | **1.24** | **0.47** |
| E2R3X4 | Occludin | OCLN | **37.78** | **0.72** | **0.23** |
| Q2KM15 | 2'-5' oligoadenylate synthetase-like 1 | Oasl1 | **4.78** | **0.97** | **11.21** |
| Q2KKD1 | 2'-5' oligoadenylate synthetase 3 | OAS3 | **8.80** | **5.85** | **4.61** |
| Q2KM16 | 2'-5' oligoadenylate synthetase 2 | Oas2 | **6.94** | **7.17** | **3.63** |
| Q2TJA4 | 2'-5' oligoadenylate synthetase 1 | OAS1 | **13.36** | **7.05** | **3.01** |
| F1PMP1 | Guanylate cyclase | NPR1 | **0.79** | **0.46** | **0.47** |
| Q9TU19 | Nephrocystin-1 | NPHP1 | **0.66** | **5.69** | **5.27** |
| A7E3K7 | Predicted NADPH oxidase-2 | Nox2 | **0.81** | **0.66** | **0.12** |
| E2QYN2 | Nuclear factor 1 | NFIX | **0.74** | **1.06** | **0.19** |
| E2REM6 | Nuclear factor 1 | NFIB | **0.99** | **1.35** | **0.27** |
| E2RMB9 | N-Myc downstream regulated gene 1 | NDRG1 | **0.74** | **0.97** | **0.33** |
| Q9N0Y2 | Interferon-induced GTP-binding protein Mx2 | MX2 | **17.50** | **10.33** | **2.42** |
| Q9N0Y3 | Interferon-induced GTP-binding protein Mx1 | MX1 | **37.78** | **30.10** | **9.09** |
| I2FJU3 | 55 kDa erythrocyte membrane protein | MPP1 | **0.66** | **0.93** | **2.53** |
| F1PYF5 | Matrix metalloproteinase-9 | MMP9 | **0.63** | **1.06** | **2.20** |
| F5C3N2 | Neprilysin-749 | MME | **2.69** | **0.88** | **2.44** |
| F1PQ35 | Malic enzyme | ME3 | **0.74** | **0.99** | **0.49** |
| J9NZX7 | Malic enzyme | ME1 | **1.10** | **0.91** | **2.95** |
| O02812 | Mitogen-activated protein kinase 14 | MAPK14 | **0.82** | **1.23** | **3.55** |
| F1PP76 | Mitogen-activated protein kinase kinase kinase kinase | MAP4K2 | **0.41** | **0.75** | **0.49** |
| E2R041 | Mitogen-activated protein kinase kinase kinase kinase | MAP4K1 | **0.68** | **1.08** | **0.20** |
| F1PCZ5 | Microtubule-associated protein | MAP4 | **0.98** | **0.92** | **0.43** |
| E2QSB9 | Microtubule-associated protein | MAP2 | **2.94** | **0.96** | **0.17** |
| Q7YRB7 | Amine oxidase [flavin-containing] B | MAOB | **0.58** | **0.16** | **0.13** |
| P58027 | Amine oxidase [flavin-containing] A | MAOA | **1.13** | **0.79** | **6.86** |
| J9P6S8 | Histone H2B | LOC48830 | **0.29** | **0.65** | **3.16** |
| H9GW87 | Transaldolase | LOC10085 | **0.88** | **0.58** | **2.52** |
| F6Y713 | Alpha-1-acid glycoprotein | LOC10068 | **5.32** | **4.73** | **2.53** |
| E2QXS1 | Lipase | LIPA | **0.74** | **0.83** | **0.18** |
| J9P3N4 | Galectin | LGALSL | **1.01** | **0.82** | **3.09** |
| F6PME1 | Galectin | LGALS3 | **0.49** | **0.74** | **2.46** |
| J9P5U3 | Galectin | LGALS1 | **0.63** | **0.66** | **3.14** |
| E2R761 | L-lactate dehydrogenase | LDHB | **3.63** | **0.82** | **0.25** |
| F1PVW0 | L-lactate dehydrogenase | LDHA | **0.80** | **0.86** | **2.13** |
| Q30344 | Integral membrane glycoprotein | LA-DRB | **1.06** | **0.92** | **17.94** |
| F1PTY1 | Keratin, type II cytoskeletal 1 | KRT1 | **0.75** | **0.60** | **2.05** |
| F1PUW9 | Mast/stem cell growth factor receptor Kit | KIT | **0.72** | **0.78** | **2.10** |
| Q9TU04 | Integrin beta | ITGB2 | **1.11** | **4.51** | **3.75** |
| E2RT60 | Integrin beta | ITGB1 | **0.86** | **0.61** | **0.35** |
| Q29467 | Type I inositol 1,4,5-trisphosphate 5-phosphatase | INPP5A | **0.70** | **0.74** | **10.51** |
| Q9BEH0 | Interleukin-1 receptor antagonist protein | IL1RN | **0.62** | **3.02** | **12.38** |
| J9NY18 | Interleukin-18 | IL18 | **4.58** | **0.54** | **2.77** |
| F1PXU6 | Tyrosine-protein kinase receptor | IGF1R | **0.70** | **1.70** | **2.14** |
| J7NP21 | Interferon-gamma-inducible GTPase IFGGB2 protein | IFGGB2 | **6.35** | **8.09** | **2.71** |
| J7PCK9 | Interferon-gamma-inducible GTPase IFGGB1 protein | IFGGB1 | **4.58** | **5.37** | **2.55** |
| F1PB95 | Intercellular adhesion molecule 1 | ICAM1 | **5.18** | **0.98** | **0.40** |
| E2R560 | Hyaluronidase | HYAL2 | **0.66** | **30.10** | **0.24** |
| F1PYE3 | Heat shock protein beta-1 | HSPB1 | **0.52** | **0.52** | **2.15** |
| F1P8N6 | Endoplasmin | HSP90B1 | **0.99** | **1.02** | **0.37** |
| E2QX67 | 15-hydroxyprostaglandin dehydrogenase | HPGD | **0.33** | **0.69** | **0.17** |
| G1K2D9 | Haptoglobin | HP | **20.30** | **15.67** | **13.16** |
| Q6YKA4 | High mobility group protein B1 | HMGB1 | **0.44** | **0.15** | **0.09** |
| E2RIM8 | Beta-hexosaminidase | HEXA | **0.52** | **0.58** | **0.32** |
| F1PR63 | Histone deacetylase | HDAC2 | **0.76** | **0.74** | **0.50** |
| P60524 | Hemoglobin subunit beta | HBB | **0.62** | **0.75** | **6.96** |
| E2RQX0 | Histone H2A | H2AFY2 | **0.73** | **0.33** | **0.35** |
| Q4ZHR9 | Guanylate cyclase soluble subunit beta-1 | GUCY1B3 | **1.00** | **1.29** | **0.67** |
| J9P0V7 | Glutathione S-transferase | GSTA3 | **0.27** | **0.66** | **3.07** |
| J9P028 | Glutathione peroxidase | GPX3 | **0.93** | **0.94** | **3.02** |
| F1Q0Z4 | Glutathione peroxidase | GPX2 | **0.94** | **1.15** | **2.25** |
| O97702 | Integrin beta | GPIIIa | **0.38** | **0.96** | **4.05** |
| E2R2C3 | Glucose-6-phosphate isomerase | GPI | **0.83** | **0.61** | **0.22** |
| J9PAK7 | Guanine nucleotide-binding protein subunit gamma | GNG2 | **0.81** | **1.26** | **3.38** |
| J9P702 | Guanine nucleotide-binding protein subunit gamma | GNG12 | **1.49** | **0.58** | **3.17** |
| P63091 | Guanine nucleotide-binding protein G(s) subunit alpha | GNAS | **1.41** | **0.70** | **6.99** |
| P38400 | Guanine nucleotide-binding protein G(i) subunit alpha-2 | GNAI2 | **1.02** | **0.43** | **5.64** |
| P52206 | Guanine nucleotide-binding protein subunit alpha-11 | GNA11 | **0.74** | **9.92** | **6.38** |
| E2RMT7 | GMP reductase | GMPR | **0.94** | **0.80** | **0.33** |
| F1Q2N5 | Glutamate dehydrogenase | GLUD1 | **0.59** | **0.26** | **2.32** |
| Q6S9C0 | Gap junction alpha-1 protein | GJA1 | **14.27** | **0.06** | **0.71** |
| F1PTZ9 | Glyceraldehyde-3-phosphate dehydrogenase | GAPDH | **0.61** | **0.55** | **2.07** |
| E2R0I9 | Glucose-6-phosphate 1-dehydrogenase | G6PD | **0.67** | **0.95** | **0.20** |
| F1PKS7 | Phospholemman | FXYD1 | **0.35** | **0.76** | **0.45** |
| F1PAF0 | Tissue alpha-L-fucosidase | FUCA1 | **0.72** | **1.00** | **0.39** |
| Q53VB8 | Ferritin light chain | FTL | **0.76** | **1.23** | **1.01** |
| Q95MP7 | Ferritin heavy chain | FTH1 | **1.68** | **0.32** | **1.51** |
| Q95MP7 | Ferritin heavy chain | FTH1 | **0.85** | **0.32** | **0.28** |
| F1PYU3 | Fascin | FSCN1 | **1.15** | **1.17** | **2.24** |
| F1P6H7 | Fibronectin | FN1 | **0.76** | **0.77** | **0.37** |
| Q95LA1 | Dimethylaniline monooxygenase [N-oxide-forming] 3 | FMO3 | **1.01** | **0.30** | **0.17** |
| E2RHC8 | Dimethylaniline monooxygenase [N-oxide-forming] | FMO2 | **0.97** | **1.12** | **0.32** |
| Q95LA2 | Dimethylaniline monooxygenase [N-oxide-forming] 1 | FMO1 | **0.99** | **0.32** | **0.63** |
| F1P9T5 | Peptidyl-prolyl cis-trans isomerase | FKBP11 | **0.08** | **0.91** | **0.38** |
| B6F2G5 | Fragile histidine triad | FHIT | **0.60** | **0.67** | **0.14** |
| F1PBL4 | Fibrinogen alpha chain | FGA | **0.93** | **0.82** | **0.41** |
| D3QXX4 | Uncharacterized protein | Escheric | **0.68** | **0.85** | **0.15** |
| J9NZE0 | Band 4.1-like protein 5 | EPB41L5 | **1.02** | **0.63** | **2.88** |
| Q8MJD1 | Neutrophil elastase | ELA2 | **0.85** | **0.28** | **0.69** |
| E2RGG6 | Eukaryotic translation initiation factor 3 subunit G | EIF3G | **1.19** | **1.34** | **0.31** |
| E2REK3 | Eukaryotic translation initiation factor 3 subunit D | EIF3D | **0.90** | **1.16** | **0.26** |
| Q2KM13 | Eukaryotic translation initiation factor 2-alpha kinase 2 | EIF2AK2 | **6.08** | **3.69** | **2.73** |
| J9P7T2 | D-tyrosyl-tRNA(Tyr) deacylase | DTD1 | **0.81** | **0.56** | **3.35** |
| E2R4Y3 | Deoxyhypusine hydroxylase | DOHH | **0.68** | **1.09** | **0.24** |
| F1P7G4 | Dystrophin | DMD | **0.61** | **0.57** | **0.37** |
| F1PAR0 | Dihydrolipoyl dehydrogenase | DLD | **1.10** | **0.72** | **0.39** |
| Q5W425 | MHC class II antigen DR alpha | DLA-DRA1 | **0.80** | **0.78** | **8.77** |
| O78116 | MHC class II DLA DQ beta chain | DLA-DQB | **1.86** | **0.74** | **3.77** |
| Q5TJG8 | MHC class II antigen DM alpha | DLA-DMA | **0.98** | **1.03** | **0.82** |
| G3DTQ8 | MHC class I antigen | DLA-88 | **0.96** | **0.80** | **2.49** |
| A0MZ32 | MHC class I antigen | DLA-88 | **1.16** | **1.04** | **0.09** |
| O98082 | MHC class I DLA-88 | dla88 | **1.05** | **0.68** | **4.17** |
| Q30410 | MHC class II DLA DQalpha chain | DLA DQA | **0.02** | **3.41** | **9.81** |
| Q5XFN2 | Desmin | DES | **0.59** | **0.64** | **7.08** |
| Q5WR10 | Spliceosome RNA helicase DDX39B | DDX39B | **0.56** | **0.82** | **0.87** |
| Q29393 | Decorin | DCN | **0.96** | **2.46** | **10.51** |
| Q9TSZ6 | Dystroglycan | DAG1 | **0.48** | **4.12** | **0.66** |
| P24460 | Cytochrome P450 2B11 | CYP2B11 | **0.39** | **0.49** | **4.88** |
| Q307K8 | Cytochrome P450 2A13 | CYP2A13 | **3.87** | **3.50** | **0.71** |
| F1P8R7 | Cytochrome P450 1A1 | CYP1A1 | **0.77** | **1.11** | **0.38** |
| P00011 | Cytochrome c | CYCS | **0.67** | **1.00** | **4.28** |
| J9P2E3 | NADH-cytochrome b5 reductase 3 | CYB5R3 | **0.95** | **1.10** | **3.08** |
| F1PAK0 | Cathepsin S | CTSS | **0.63** | **0.44** | **0.39** |
| Q4LAL9 | Cathepsin D | CTSD | **0.69** | **0.71** | **7.51** |
| O97578 | Dipeptidyl peptidase 1 | CTSC | **2.24** | **0.87** | **4.01** |
| E2QUQ3 | CTP synthase | CTPS1 | **0.64** | **0.64** | **0.17** |
| B6V8E6 | Catenin beta-1 | CTNNB1 | **1.38** | **0.46** | **0.14** |
| T2KEN6 | C-reactive protein | CRP | **21.03** | **0.83** | **1.21** |
| J9P549 | Cytochrome c oxidase subunit 7A2, mitochondrial | COX7A2 | **0.52** | **1.80** | **3.11** |
| E2REL6 | Coronin | CORO2B | **0.58** | **0.76** | **0.26** |
| E2QSZ5 | Coronin | CORO1A | **0.90** | **0.84** | **0.17** |
| F1PU93 | Coatomer subunit alpha | COPA | **0.74** | **0.86** | **2.07** |
| F1PHY1 | Collagen alpha-2(I) chain | COL1A2 | **0.93** | **0.92** | **0.45** |
| F1Q3I5 | Collagen alpha-1(I) chain | COL1A1 | **0.98** | **0.99** | **2.34** |
| P21842 | Chymase | CMA1 | **0.30** | **0.54** | **4.84** |
| P05123 | Creatine kinase M-type | CKM | **0.83** | **0.59** | **4.55** |
| D3YJ60 | Chitinase 3-like 1 | CHI3L1 | **0.64** | **0.56** | **0.17** |
| A7YH32 | Cingulin | CGN | **0.54** | **0.83** | **0.13** |
| Q95N05 | Carboxylesterase D1 | CESdD1 | **0.59** | **0.32** | **0.42** |
| Q004B2 | Carcinoembryonic antigen-related cell adhesion molecule 1 isoform 4L | CEACAM1 | **0.92** | **0.72** | **7.12** |
| F1Q2H0 | Phosphatidate cytidylyltransferase | CDS1 | **0.10** | **0.70** | **2.31** |
| F1PAA4 | Cadherin-1 | CDH1 | **0.80** | **0.93** | **0.38** |
| Q2Q423 | CD97 large isoform | CD97 | **1.46** | **0.88** | **13.41** |
| P33705 | T-cell surface glycoprotein CD4 | CD4 | **0.94** | **5.02** | **5.42** |
| D5IGC7 | CD36 antigen | CD36 | **5.66** | **2.96** | **0.17** |
| F1PL59 | Scavenger receptor cysteine-rich type 1 protein M130 | CD163 | **0.50** | **0.99** | **0.47** |
| P33724 | Caveolin-1 | CAV1 | **0.63** | **0.56** | **5.47** |
| O97492 | Catalase | CAT | **0.96** | **0.90** | **3.96** |
| Q45T68 | Caspase | CASP9 | **0.15** | **0.22** | **0.69** |
| Q38JA9 | Caspase 8 | CASP8 | **0.58** | **4.97** | **19.38** |
| G1K2D5 | Calcyphosin | CAPS | **0.27** | **0.28** | **0.21** |
| E2RH53 | Adenylyl cyclase-associated protein | CAP2 | **1.00** | **0.72** | **0.31** |
| E2QZ50 | Adenylyl cyclase-associated protein | CAP1 | **0.57** | **0.49** | **0.20** |
| P24643 | Calnexin | CANX | **0.67** | **0.75** | **4.90** |
| Q6TN20 | Cathelicidin | CAMP | **0.88** | **0.14** | **0.21** |
| J9P0L8 | C5a anaphylatoxin chemotactic receptor 1 | C5AR1 | **4.95** | **0.83** | **3.05** |
| G1K2D8 | Biglycan | BGN | **0.44** | **0.66** | **2.49** |
| Q76LT7 | Bcl-xL | bcl-xL | **4.44** | **0.93** | **2.02** |
| F1PY28 | Branched-chain-amino-acid aminotransferase | BCAT1 | **0.74** | **0.24** | **2.14** |
| E2RN10 | Beta-2-microglobulin | B2M | **4.96** | **4.90** | **2.61** |
| J9NZQ3 | V-type proton ATPase subunit G 1 | ATP6V1G1 | **1.28** | **0.84** | **2.92** |
| E2RN91 | V-type proton ATPase subunit F | ATP6V1F | **0.98** | **0.75** | **0.33** |
| E2RF46 | Asparagine synthetase | ASNS | **0.98** | **0.77** | **0.28** |
| E2RD65 | Actin-related protein 2/3 complex subunit 5 | ARPC5 | **1.25** | **0.71** | **0.26** |
| J9NV13 | AT-rich interactive domain-containing protein 5B | ARID5B | **0.62** | **0.80** | **2.62** |
| F1PJ74 | Apolipoprotein E | APOE | **5.80** | **4.65** | **2.82** |
| J9NWJ6 | Apolipoprotein C-II | APOC2 | **1.16** | **0.82** | **2.73** |
| J9NS21 | Apoptotic protease-activating factor 1 | APAF1 | **0.60** | **0.95** | **2.61** |
| E2RFB7 | Amine oxidase | AOC3 | **1.06** | **0.94** | **0.28** |
| E2R0S6 | Annexin | ANXA8L1 | **0.74** | **1.22** | **0.22** |
| F1PXG4 | Annexin | ANXA4 | **0.55** | **1.44** | **2.14** |
| Q6TEQ7 | Annexin A2 | ANXA2 | **0.63** | **0.11** | **0.15** |
| F1P6B7 | Annexin | ANXA1 | **0.70** | **0.02** | **0.36** |
| F1PCB5 | Aminopeptidase N | AN | **1.72** | **0.79** | **0.41** |
| F1PS82 | Fructose-bisphosphate aldolase | ALDOC | **0.69** | **0.76** | **2.03** |
| F1PBT3 | Fructose-bisphosphate aldolase | ALDOA | **0.80** | **0.11** | **0.41** |
| E2QZ39 | Aldehyde dehydrogenase | ALDH3B1 | **0.95** | **0.87** | **0.20** |
| F1PDR0 | CD166 antigen | ALCAM | **0.77** | **0.81** | **0.43** |
| P49822 | Serum albumin | ALB | **0.67** | **0.53** | **5.69** |
| F2Z4Q6 | Serum albumin | ALB | **0.46** | **0.58** | **2.44** |
| B4YY02 | Adenylate kinase 1 | AK1 | **0.70** | **0.88** | **0.13** |
| E2REN0 | Adenosylhomocysteinase | AHCYL1 | **0.87** | **0.93** | **0.28** |
| F1PBL3 | Glycogen debranching enzyme | AGL | **0.82** | **1.04** | **0.40** |
| F1Q4F5 | Adenylosuccinate synthetase isozyme 2 | ADSS | **0.63** | **0.49** | **2.40** |
| Q28343 | Aggrecan core protein | ACAN | **2.90** | **0.50** | **10.20** |
| F1P699 | Phosphatidylethanolamine-binding protein 1 |  | **0.67** | **1.29** | **0.36** |
| Q9N0V0 | Alkaline phosphatase |  | **0.94** | **3.30** | **2.63** |
| Q5JCF5 | Utrophin |  | **0.53** | **0.54** | **0.80** |
| Q3HTU3 | Angiotensin I converting enzyme 1 |  | **1.08** | **0.77** | **0.22** |
| Q3HTT6 | Hydroxysteroid 11-beta dehydrogenase 2 |  | **1.31** | **0.78** | **3.09** |
| Q3HTT4 | Endothelin converting enzyme-1 |  | **2.19** | **2.02** | **24.34** |
| J9P9U4 | Reticulon |  | **0.69** | **0.84** | **3.38** |
| F1PCH3 | Enolase |  | **0.41** | **1.00** | **0.42** |
| E7ECW0 | Arachidonate 5-lipoxygenase |  | **0.71** | **0.96** | **0.36** |
| E2R4Z2 | Coronin |  | **0.56** | **0.49** | **0.24** |
| A1ILJ0 | Alpha 1 antitrypsin |  | **2.36** | **0.99** | **0.10** |

Red:up-regulation (≥2);

Gray: No significat difference;

Green:down-regulation(≤0.5)
